# Isolation of Non-Tuberculous Mycobacteria in Children Investigated for Pulmonary Tuberculosis

**DOI:** 10.1371/journal.pone.0000021

**Published:** 2006-12-20

**Authors:** Mark Hatherill, Tony Hawkridge, Andrew Whitelaw, Michele Tameris, Hassan Mahomed, Sizulu Moyo, Willem Hanekom, Gregory Hussey

**Affiliations:** 1 South African Tuberculosis Vaccine Initiative (SATVI), Institute of Infectious Disease and Molecular Medicine, University of Cape Town Cape Town, South Africa; 2 School of Child and Adolescent Health, University of Cape Town Cape Town, South Africa; 3 Department of Clinical Laboratory Sciences, University of Cape Town Cape Town, South Africa; 4 National Health Laboratory Service Cape Town, South Africa; Duke University Medical Center, United States of America

## Abstract

**Objective:**

To evaluate the frequency and clinical significance of non-tuberculous mycobacteria (NTM) isolates among children investigated for pulmonary tuberculosis in a rural South African community.

**Methods:**

Children were investigated for pulmonary tuberculosis as part of a tuberculosis vaccine surveillance program (2001–2005). The clinical features of children in whom NTM were isolated, from induced sputum or gastric lavage, were compared to those with culture-proven *M. tuberculosis*.

**Results:**

Mycobacterial culture demonstrated 114 NTM isolates from 109 of the 1,732 children investigated, a crude yield of 6% (95% CI 5–7). The comparative yield of positive NTM cultures from gastric lavage was 40% (95% CI 31–50), compared to 67% (95% CI 58–76) from induced sputum. 95% of children with NTM isolates were symptomatic. Two children were HIV-infected. By contrast, *M. tuberculosis* was isolated in 187 children, a crude yield of 11% (95% CI 9–12). Compared to those with culture-proven *M. tuberculosis*, children with NTM isolates were less likely to demonstrate acid-fast bacilli on direct smear microscopy (OR 0.19; 95% 0.0–0.76). Children with NTM were older (p<0.0001), and more likely to demonstrate constitutional symptoms (p = 0.001), including fever (p = 0.003) and loss of weight or failure to gain weight (p = 0.04), but less likely to demonstrate a strongly positive tuberculin skin test (p<0.0001) or radiological features consistent with pulmonary tuberculosis (p = 0.04).

**Discussion:**

NTM were isolated in 6% of all children investigated for pulmonary tuberculosis and in more than one third of those with a positive mycobacterial culture. NTM may complicate the diagnosis of PTB in regions that lack capacity for mycobacterial species identification. The association of NTM isolates with constitutional symptoms suggestive of host recognition requires further investigation.

## Introduction

The diagnosis of pulmonary tuberculosis (PTB) in children is difficult, since disease is often pauci-bacillary [1]. Bacteriological confirmation of *M. tuberculosis* in children may be possible in fewer than 40% of suspect cases, yet it remains the gold standard in the majority of diagnostic approaches [1]. In practice, high-prevalence, resource-limited countries rely on history-taking, tuberculin skin testing, and chest radiography, for investigation of PTB in children [1]. Gastric lavage or induced sputum sampling may not be feasible in many developing country settings, and even if smear microscopy is performed, mycobacterial culture or PCR for species identification may not be available, with the result that a diagnosis of *M. tuberculosis* will be made if any acid-fast bacilli are detected [1].

Non-tuberculous mycobacteria (NTM) are ubiquitous environmental acid-fast bacilli, which are present in water, soil, and biofilms [2]. Since NTM are resistant to chlorine and biocide disinfection, they frequently contaminate municipal drinking water distribution systems [2]–[3]. Although NTM are not obligate pathogens, they share the features of hardiness, hydrophobicity, aerosolization, and intracellular pathogenicity, with *M. tuberculosis* [2]. The probability of NTM disease is likely to increase with the extent of environmental exposure, particularly in farming communities, with immune susceptibility to opportunistic infection, and with co-existent chronic lung disease such as cystic fribrosis [2]–[4]. However, a recent review of the English literature over a period of 70 years describes 43 cases of pulmonary disease caused by NTM in previously well children [5]. In contrast, cervical lymphadenitis is a relatively common mode of presentation in childhood [6]. Increased awareness of NTM, and improved methods of detection, may make it increasingly difficult to distinguish NTM disease from environmental colonization [2].

It might also be difficult to distinguish tuberculous from non-tuberculous mycobacterial lung disease on the basis of clinical and radiological features [5]. Detection of non-tuberculous acid-fast bacilli in respiratory secretions by direct smear microscopy might be misinterpreted as PTB unless mycobacterial species identification is available [7]. In this region, the relative prevalence of *M. tuberculosis* and NTM in respiratory secretions, and therefore the magnitude of this diagnostic dilemma in the diagnosis of childhood PTB, remains unknown [1].

These issues are clearly important for the diagnosis and treatment of childhood tuberculosis, but are also crucial in the design of precise clinical and microbiological endpoints for trials of novel tuberculosis vaccines in these regions. Meta-analyses of previous trials of bacille Calmette-Guerin (BCG) vaccination concluded that protection due to BCG was better in trials reporting laboratory-confirmed cases of tuberculosis and that diagnostic error may lead to under-estimation of vaccine efficacy [8]. It has also been suggested that greater exposure to environmental mycobacterial antigens in tropical and sub-tropical latitudes might be responsible for the reduced efficacy of BCG vaccine trials in these regions [9]. We aimed to determine the frequency and clinical significance of NTM isolates among children investigated for PTB at a tuberculosis vaccine trial site in a rural South African community.

## Methods

This community-based study was carried out at a tuberculosis vaccine trial site in a rural area 100 km outside Cape Town, South Africa. The region is a wheat, fruit, and sheep farming area with a population of approximately 350 000, serviced by 4 primary and secondary level hospitals and a network of day clinics. Almost 90% of households have formal housing, 70% are supplied with electricity, and 65% are supplied with sanitation and piped water [10]. In 2003, the infant mortality rate was 28 per 1000 live births and the incidence of PTB was 583 per 100 000 [10]. In the South African context, the antenatal Human Immunodeficiency Virus (HIV) sero-prevalence rate has been relatively low at 8% [10].

Children with possible PTB were prospectively identified during surveillance of 11677 children enrolled in an ongoing phase 4 BCG vaccine trial (2001–2005). Passive surveillance included perusal of hospital admission records, radiology reports, tuberculosis registers, and communication with district clinic staff throughout the study area. Active surveillance was not conducted and therefore data from children who did not come into contact with the health services, or tuberculosis control programme, were not collected. Indications for investigation included a known adult contact with PTB, or recent attendance at public sector health services with an illness consistent with PTB. Children were then admitted to the trial Case Verification Ward at the regional tuberculosis hospital for diagnostic purposes, with the written informed consent of the parent or legal guardian. Subsequent out-patient treatment for PTB, if indicated, was managed by the regional tuberculosis control programme, with a follow-up visit to evaluate adherence to treatment. The trial was approved by the Research Ethics Committee of the University of Cape Town and monitored by a Data Safety Monitoring Board (DSMB).

The following data were prospectively collected: age; weight; a history of fever, night sweats, recent loss of weight, cough, or wheeze; and respiratory rate, lymphadenopathy, or lung crepitations, on clinical examination. Failure to gain weight was defined as crossing of weight-for-age centiles on the Road-To-Health card. Weight-for-age Z scores were calculated using Epi Info™ Version 3.3.2 (Centers for Disease Control and Prevention, Department of Health and Human Services, USA), based upon the 2000 CDC reference values. Percentage oxygen saturation was measured by pulse oximetry. Mantoux and Tine tuberculin skin tests were performed in all children, as well as a chest radiograph, and a rapid HIV test (with confirmatory HIV ELISA and PCR, if positive). The chest radiograph was graded by an experienced tuberculosis clinician, primarily on the basis of hilar or paratracheal lymphadenopathy, with or without atelectasis or consolidation, as either ‘normal’; ‘abnormal, but not PTB’; ‘suspicious of PTB’; or ‘likely to be PTB’. For the purpose of comparison, these four categories were further compressed to a binary variable, either ‘suggestive’ or ‘not suggestive’ of PTB.

Two gastric lavage and two induced sputum samples were obtained on consecutive days, using a modified method, previously described, which has been shown to be effective in infants and children less than 2 years of age [11]. Sterile single-use consumables were used for each clinical procedure. Laboratory procedures were carried out at an accredited laboratory (South African National Accreditation Service) with international quality control provided by the National External Quality Assurance Service (NEQAS). Specimens were decontaminated using standard laboratory procedures. Briefly, specimens were mixed with equal volumes of 6% NaOH/2,9% Na citrate and N-acetyl cysteine and left for 15 minutes. Neutralization was achieved by the addition of phosphate buffered saline (pH 6,8). These were then centrifuged for 15 minutes, the supernatant discarded, and the deposit re-suspended in phosphate buffer. A smear was prepared from this deposit and stained using the auramine O fluorescent stain. An aliquot of the deposit was inoculated into a Mycobacterial Growth Indicator Tube (MGIT) according to the manufacturer's instructions (Becton Dickinson and Company, Sparks, Md, USA), and onto Lowenstein-Jensen agar slopes. Cultures were incubated for 8 weeks before being recorded as negative.

Positive cultures were screened by Ziehl-Neelsen and Gram stain. Those cultures showing acid-fast bacilli were identified as *Mycobacterium tuberculosis* complex by the Accuprobe *M. tuberculosis* complex culture identification test, according to the manufacturer's instructions (Gen-Probe, San Diego, Ca, USA). Those cultures positively identified by the Accuprobe test were further identified by a PCR assay to differentiate *M. bovis* BCG from other members of the *M. tuberculosis* complex [12].

If the Accuprobe assay was negative, isolates were identified as *M. tuberculosis* by PCR as previously described [13]. Amplification of a 336bp band using this assay served to identify the organism as *M. tuberculosis*. Cultures showing no amplification with this PCR assay were further identified by a second assay as described by Telenti et al and modified by Brunello et al [14]–[15]. Briefly, a portion of the *hsp*65 gene was amplified, and the resultant 439bp product digested by the restriction enzymes *Bst*EII and *Hae*III. The sizes of the fragments generated were used to identify the organisms to species level. If the organism morphologically appeared to be one of the other aerobic actinomycetes, and had not been identified by the restriction enzymes as described above, the *hsp*65 amplicon was digested with *Msp*I and *Hinf*I, and the identity of the organism determined by using the algorithm described by Steingrube et al [16].

The crude yield of mycobacterial culture for NTM was calculated as a proportion of all children who underwent investigation. The comparative yield of NTM culture from gastric lavage and induced sputum was calculated as a proportion of all children from whom NTM were isolated. Children from whom both NTM and *M. tuberculosis* were isolated were included in the comparison of diagnostic yields from either gastric lavage or induced sputum, but were excluded from the comparison of symptoms and clinical signs associated with either NTM or *M. tuberculosis*.

Reports of positive culture results and a summary of clinical findings and investigations were provided to the regional health services and tuberculosis control programme for ongoing management. Children in whom PTB was diagnosed were routinely treated for 6 months (2 months rifampicin, isoniazid, and pyrazinamide; 4 months rifampicin and isoniazid) and followed to completion of treatment by means of a clinic register. During the data collection period, NTM were thought to be environmental contaminants in children without immune deficiency or underlying chronic lung disease, rather than pathogens, and therefore specific antibiotic therapy for NTM was not routinely recommended. In-patient records of all subsequent hospital admissions for respiratory illness were examined and a study follow-up visit was conducted for children in whom PTB was diagnosed, or in whom an NTM was isolated. Records of out-patient clinic attendances, general practitioner visits, and antibiotic prescription records were not collected by the surveillance system.

Data are presented as n (%), 95% confidence intervals (95% CI) for proportions, and median and inter-quartile range (IQR) for continuous data. Categorical data were analyzed by the Fisher's Exact test or Chi-squared test, as appropriate, and continuous data were analyzed by the Mann-Whitney test, using Analyse-It statistical software (Analyse-It, UK).

## Results

Over a period of 4 years (2001–2005), 1732 children (15%) had been admitted for investigation at the time of analysis. Mycobacterial culture had yielded 72 identifiable NTM isolates, comprising 8 different species, and 37 non-identifiable NTM, from 215 gastric lavage and 214 induced sputum specimens, in 109 children (see Table 1). Initial direct smear microscopy had demonstrated scanty acid-fast bacilli in two children. All gastric lavage and induced sputum specimens from the remaining 107 children had been direct smear microscopy negative. Two or more NTM species were isolated in 4 children, and 5 children were co-infected with *M. tuberculosis*.

**Table 1:**
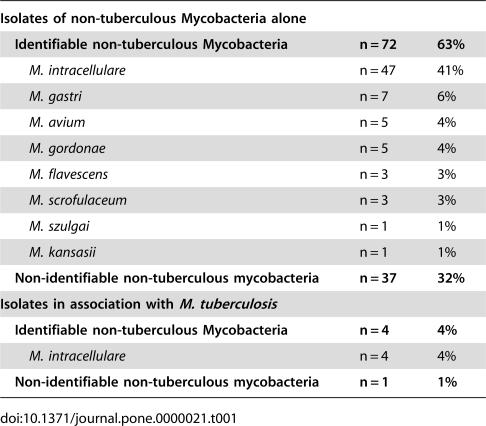
Non-tuberculous mycobacterial isolates (n = 114) obtained from gastric lavage or induced sputum specimens in children investigated for tuberculosis (n = 109).

**Isolates of non-tuberculous Mycobacteria alone**		
**Identifiable non-tuberculous Mycobacteria**	**n = 72**	**63%**
*M. intracellulare*	n = 47	41%
*M. gastri*	n = 7	6%
*M. avium*	n = 5	4%
*M. gordonae*	n = 5	4%
*M. flavescens*	n = 3	3%
*M. scrofulaceum*	n = 3	3%
*M. szulgai*	n = 1	1%
*M. kansasii*	n = 1	1%
**Non-identifiable non-tuberculous mycobacteria**	**n = 37**	**32%**
**Isolates in association with ** ***M. tuberculosis***		
**Identifiable non-tuberculous Mycobacteria**	**n = 4**	**4%**
*M. intracellulare*	n = 4	4%
**Non-identifiable non-tuberculous mycobacteria**	**n = 1**	**1%**

The crude yield of mycobacterial culture for NTM (n = 109) was 6% (95% CI 5–7) overall; 3% (95% CI 2–3) from gastric lavage (n = 44); and 4% (95% CI 3–5) from induced sputum (n = 73). NTM were demonstrated by both gastric lavage and induced sputum in 8 children. The comparative yield of NTM culture (n = 109) was 40% (95% CI 31–50) for gastric lavage (n = 44), compared to 67% (95% CI 58–76) for induced sputum (n = 73). The comparative yield of NTM culture for the two most common species, *M. intracellulare* (n = 47) and *M. gastri* (n = 7), was 30% (95% CI 17–43) and 57% (95% CI 20–94) for gastric lavage, compared to 74% (95% CI 62–87) and 71% (95% CI 38–100) for induced sputum.

**Figure 1:**
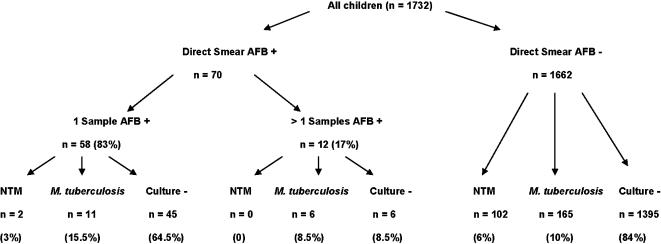
Distribution of children with NTM (n = 104) or *M. tuberculosis* (n = 182) isolates according to whether acid-fast bacilli (AFB) were detected on initial direct smear microscopy. Children with dual NTM and *M. tuberculosis* isolates (n = 5) are excluded. Proportions are presented as a percentage of Direct Smear AFB+(n = 70) or Direct Smear AFB–children (n = 1662). Data are n (%).

For comparison, the crude yield of mycobacterial culture for *M. tuberculosis* (n = 187) was 11% (95% CI 9–12), resulting in a yield ratio of tuberculous to non-tuberculous mycobacteria of less than 2:1. An additional 51 children (3%; 95% CI 2–4) had been positive or scanty positive for acid-fast bacilli on direct smear microscopy, but subsequently mycobacterial culture and PCR negative. Among children with a positive mycobacterial culture, those with NTM isolates were less likely to have had acid-fast bacilli detected on initial direct smear microscopy, odds ratio 0.19 (95% CI 0.0–0.76), than those with *M. tuberculosis*. See Figure 1.

### Clinical data in children with NTM isolates

The clinical features of children from whom NTM alone were isolated (n = 104) are summarized in Table 2. Only 4 children (4%) had lymphadenopathy, which was cervical and without adenitis in all cases. Median baseline respiratory rate was 28 per minute (26–30). Respiratory symptoms and signs included cough (n = 58; 56%), wheeze (n = 51; 49%); and lung crepitations (n = 26; 25%), but no children demonstrated sub-costal recession. Only 5 children (5%) were entirely well, without any constitutional or respiratory symptoms or signs.

**Table 2:**
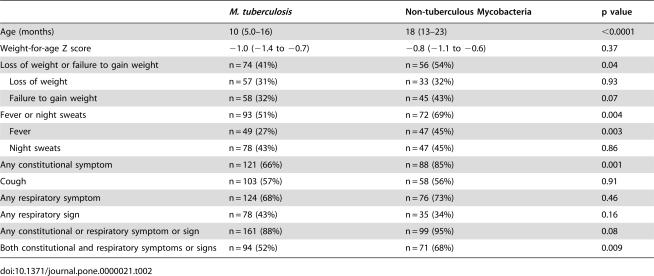
Comparison of demographic and clinical features in children from whom either *M. tuberculosis*, (n = 182) or non-tuberculous Mycobacteria (n = 104) were isolated. Children with dual NTM and *M. tuberculosis* isolates (n = 5) are excluded. Data are n (%) or median (IQR).

	*M. tuberculosis*	Non-tuberculous Mycobacteria	p value
Age (months)	10 (5.0–16)	18 (13–23)	<0.0001
Weight-for-age Z score	−1.0 (−1.4 to −0.7)	−0.8 (−1.1 to −0.6)	0.37
Loss of weight or failure to gain weight	n = 74 (41%)	n = 56 (54%)	0.04
Loss of weight	n = 57 (31%)	n = 33 (32%)	0.93
Failure to gain weight	n = 58 (32%)	n = 45 (43%)	0.07
Fever or night sweats	n = 93 (51%)	n = 72 (69%)	0.004
Fever	n = 49 (27%)	n = 47 (45%)	0.003
Night sweats	n = 78 (43%)	n = 47 (45%)	0.86
Any constitutional symptom	n = 121 (66%)	n = 88 (85%)	0.001
Cough	n = 103 (57%)	n = 58 (56%)	0.91
Any respiratory symptom	n = 124 (68%)	n = 76 (73%)	0.46
Any respiratory sign	n = 78 (43%)	n = 35 (34%)	0.16
Any constitutional or respiratory symptom or sign	n = 161 (88%)	n = 99 (95%)	0.08
Both constitutional and respiratory symptoms or signs	n = 94 (52%)	n = 71 (68%)	0.009

Median baseline oxygen saturation measured by pulse oximetry was 100% (99–100). A total of 31 children (30%) demonstrated either a strongly positive Mantoux (>/ = 15 mm), or Tine reaction (Grade 3 or 4). Ninety-three children (89%) were tested for HIV, of whom none were HIV-infected. An additional 2 children with NTM isolates (*M. intracellulare* and *M. gordonae* respectively) were found to have been tested and diagnosed as HIV-infected prior to admission.

At the time of ward discharge, 7 chest radiographs (7%) were classified by the treating clinician as ‘likely to be PTB’, 27 (26%) as ‘suspicious of PTB’, and 11 (11%) as ‘abnormal, but not consistent with PTB’. On the basis of the clinical and radiological findings, 46 children (44%) were presumptively diagnosed as having PTB at the time of ward discharge and were referred to the health services for anti-tuberculous therapy.

### Follow-up data in children with NTM isolates

Six children from whom NTM had been isolated were subsequently hospitalized for pneumonia (lobar pneumonia n = 3; bronchopneumonia n = 3) at intervals ranging from 2 weeks to 7 months after discharge from the Case Verification Ward. All 6 children recovered fully after treatment with either penicillin or a cephalosporin. One child, who had been presumptively diagnosed with PTB and treated with 6 months of anti-tuberculous drugs, was later hospitalized for a second time with a diagnosis of tuberculous meningitis.

Sixty-eight children with NTM isolates (62%) received a follow-up visit, of whom 42 (39%) were asymptomatic; 16 children (15%) were symptomatic (including one child with cervical lymphadenopathy) and were referred for investigation and possible treatment of suspected NTM disease; 9 children were found to have left the study area; and 1 HIV-infected child had died. No children had received in-patient or out-patient antibiotic treatment for NTM disease at the time of follow-up, although 18 (17%) of the asymptomatic and 7 (6%) of the symptomatic children had been treated for PTB.

### Comparison of children with NTM and M. tuberculosis isolates

As shown in Table 2, children with NTM isolates were older (p<0.0001), and more likely to report constitutional symptoms (p = 0.001), such as fever (p = 0.003), and either loss of weight or failure to gain weight (p = 0.04), than children from whom *M. tuberculosis* was isolated. Although children with NTM isolates did not have a higher frequency of respiratory symptoms (p = 0.46), or signs (p = 0.16), they were more likely to demonstrate a combination of both constitutional and respiratory abnormalities (p = 0.009).

**Figure 2:**
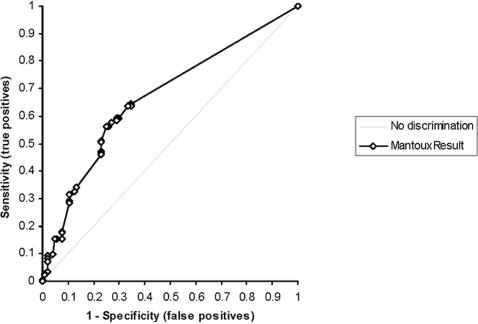
Receiver Operating Characteristic plot of Mantoux tuberculin skin test reaction (mm) for differentiation of *M. tuberculosis* from NTM isolates. Area under the curve (AUC) 0.67 (95% CI 0.60–0.73) (p<0.0001).

Sixty-eight children (65%) from whom NTM were isolated demonstrated a negative (ie. completely unreactive) Mantoux tuberculin skin test, compared to 65 children (36%) with *M. tuberculosis* isolates (p<0.0001). The median size of the reaction to the Mantoux tuberculin skin test was 0 mm (IQR 0–9) in children with NTM, compared to 12 mm (IQR 0–20) in children with *M. tuberculosis* (p<0.0001). Similarly, children with NTM isolates demonstrated a median Grade 1 (IQR 0–2) Tine reaction, compared to Grade 2 (IQR 0–4) in those with *M. tuberculosis* (p = 0.0001). The size of the Mantoux reaction demonstrated only moderate ability to discriminate *M. tuberculosis* from NTM, with area under the receiver operating characteristic (ROC) curve of 0.67 (95% CI 0.60–0.73), (p<0.001). See Figure 2.

When the classification of chest radiographs by the treating clinician was compressed to a binary variable (ie. suggestive or not suggestive of PTB), 33% of chest radiographs from children with NTM (n = 34) were suggestive of PTB, compared to 45% of chest radiographs from those with *M. tuberculosis* (n = 82), (p = 0.04).

## Discussion

We have demonstrated in a rural South African community that NTM were isolated in 6% of all children who were investigated for PTB, and in more than one third of those with a positive mycobacterial culture. This is an agricultural area and children in the community might be at greater risk of exposure to environmental NTM than their urban counterparts [2], [4]. This hypothesis is supported by the finding that children with NTM isolates were significantly older than those from whom *M. tuberculosis* was cultured, consistent with increased environmental soil exposure in older ambulant toddlers. However, the high recovery rate of NTM may also be related to the technique used to obtain clinical samples. The yield of NTM from induced sputum culture was greater than that from gastric lavage, particularly in the case of *M. intracellulare*. These findings are similar to those of a previous diagnostic study in childhood PTB, suggesting that induced sputum is a superior technique for detection of both tuberculous and non-tuberculous mycobacteria [11].

There have been few reports of NTM lung disease in otherwise well children, although cervical adenitis is relatively common in childhood [5]–[6]. We have prospectively documented isolation of NTM from respiratory secretions in more than a hundred children, the majority of whom were symptomatic. Great caution should be exercised in labeling these as cases of NTM lung disease, even though children with NTM isolates exhibited symptoms consistent with host recognition, rather than environmental colonization. The majority of the NTM species were respiratory pathogens known to cause intrathoracic disease, and previous reports of pediatric NTM disease have also documented a predominance of *M. avium intracellulare complex*, consistent with our findings [5], [6], [17]. It is notable that compared to children with microbiologically confirmed *M. tuberculosis*, those children from whom NTM were isolated were even more likely to display constitutional symptoms compatible with disease. However, although these constitutional symptoms occurred in association with respiratory symptoms or signs, children with NTM isolates were less likely to demonstrate supporting radiographic features compatible with pulmonary disease. Lastly, although the natural history of NTM disease is not known, the majority of children recovered without specific antibiotic therapy for NTM.

It may be difficult to distinguish the etiology of lung disease in children with suspected PTB, since common respiratory symptoms and signs were associated with both NTM and *M. tuberculosis* isolates. In developing regions, a presumptive diagnosis of childhood PTB is often made on the basis of clinical features, a tuberculin skin test, and chest radiography, without bacteriological confirmation [1]. However, interpretation of chest radiography may be variable, pulmonary disease caused by NTM has a similar radiographic picture to that of *M. tuberculosis*, and it has been suggested that response to tuberculin skin testing may represent partial cross-reactivity to NTM antigens [17]–[18]. Therefore, taking into account a high index of suspicion related to the regional prevalence of tuberculosis, it is not surprising that PTB had been diagnosed in many of these children before mycobacterial culture results became available.

Isolation of NTM should not rule out tuberculosis as the cause of pulmonary disease, since the constitutional symptoms associated with NTM isolates might have been due to undiagnosed, culture-negative tuberculosis. However, given the lesser reaction of children with NTM to tuberculin skin testing, this does not appear to be the case. It is also notable that only two of the children with NTM isolates were HIV-infected, which may reflect either low maternal HIV infection rates, or the success of interventions aimed at decreasing perinatal HIV transmission [10]. Although the remainder of the children was not formally investigated for immuno-deficiencies other than HIV infection, the majority were assumed to be immuno-competent.

Since clinicians in resource-limited settings rely primarily on clinical and radiological diagnostic techniques for investigation of suspected PTB in children, it would be difficult to differentiate symptomatic NTM from tuberculous disease without microbiological confirmation [1]. It is doubly unfortunate that this region, which has high prevalence rates of PTB, also appears to have high levels of exposure to environmental NTM [10]. The yield ratio of *M. tuberculosis* to NTM culture was less than 2∶1, suggesting that a diagnosis of PTB may not be assumed once acid-fast bacilli are detected in respiratory secretions. However, since children with NTM isolates were less likely to demonstrate acid-fast bacilli on direct smear microscopy, this potential diagnostic dilemma may not be relevant in practice. The identities of the acid-fast bacilli seen on smear microscopy, but which did not grow on subsequent culture, are unknown. This phenomenon may reflect vigorous decontamination during specimen handling, or inherent difficulty in isolating one or more mycobacterial species on culture.

The newer immunodiagnostic assays for *M. tuberculosis* might not completely avoid these diagnostic difficulties, since several of the organisms isolated in this study, such as *M. szulgai, M. flavescens*, and *M. gastri*, also possess RD1-encoded antigens [18]–[19]. However, the lesser reaction of children with NTM to tuberculin skin testing, and the large proportion of unreactive Mantoux tests, suggests that cross-reactivity between *M. tuberculosis* and NTM antigens may be less of a confounding issue than has been thought [18]. The data suggest that tuberculin skin testing might still be useful in differentiating children with NTM from those with *M. tuberculosis*, although with only moderate discriminatory ability.

This study has several important limitations that should be considered when interpreting the data. Children were investigated only if they were thought to be at risk of PTB, due to a suspect illness or tuberculosis contact, which introduces an ascertainment bias. Since we are unable to determine the background NTM contamination rate in a control group, we have attempted to overcome this factor by comparing only children with culture-confirmed NTM or *M. tuberculosis*, thereby excluding all those who might have had undiagnosed culture-negative disease of either aetiology. Secondly, we report subjective and objective clinical features, in association with microbiological evidence, but the radiological and histological evidence that would be required to demonstrate an association between the recovery of NTM and lung pathology is lacking. Only 2 children had been direct smear positive for acid-fast bacilli, only 8 children demonstrated NTM isolates on more than one culture, and only a third of chest radiographs were suggestive of mycobacterial lung disease, so that few of these cases would meet the American Thoracic Society criteria for the diagnosis of NTM lung disease [20]. However, since such stringent criteria would be inappropriate for the diagnosis of childhood PTB, it could equally be argued that these criteria should not be applied to the diagnosis of NTM disease in children [1].

It is also possible that decontamination procedures contributed to the high ‘smear positive culture negative’ rate and that the true rate of isolation of NTM, or *M. tuberculosis*, is even higher than that reported. Conversely, it is conceivable that laboratory contamination may have produced some false positive NTM isolates, but since 8 different NTM species were demonstrated, it is unlikely that systematic NTM contamination occurred. Finally, since follow-up surveillance collected only hospital data, we are unable to report duration of symptoms in the out-patient setting. For the above reasons, the intriguing relationship between NTM isolates and constitutional symptoms deserves further investigation, but should not be over-interpreted as a causal association with NTM disease.

Several other questions remain unanswered. The burden of environmental NTM exposure in this rural community is not known, nor is the prevalence of respiratory tract colonization among well children. This region of South Africa has one of the highest incidence rates of PTB in the world, and since it has been suggested that exposure to environmental NTM might adversely affect the protective immunity imparted by BCG vaccination, the relationship between NTM isolation and the high incidence of PTB in this region requires exploration [9], [10], [21]. In the context of future phase 3 trials of novel tuberculosis vaccines, the prevalence of mycobacteria other than *M. tuberculosis* should be taken into account when drafting the clinical, radiological, and microbiological diagnostic criteria for the primary outcome, ie. childhood tuberculosis. It remains to be seen whether the immunogenicity and efficacy of such tuberculosis vaccines might be affected by exposure to environmental NTM antigens, as in the case of BCG [9], [21]. We suggest that the rate of isolation of NTM should be analysed as a potential confounder when comparing future tuberculosis vaccine trials in different geographical areas.

In conclusion, we have demonstrated that NTM were isolated in 6% of all children investigated for PTB, and in more than a third of those with a positive mycobacterial culture, with the highest yield coming from induced sputum. Children with NTM isolates were less likely to demonstrate acid-fast bacilli on initial direct smear microscopy than those with *M. tuberculosis*. Nevertheless, the presence of NTM further complicates the diagnosis of childhood PTB in resource-limited countries with limited capacity for microbiological confirmation and mycobacterial species identification.

Children with NTM isolates were more likely to report constitutional symptoms, but demonstrated less reaction to tuberculin skin testing, than children with *M. tuberculosis*. The association of NTM isolates with symptoms suggestive of host recognition, among immuno-competent children without underlying chronic lung disease, requires further investigation, but should not be over-interpreted as NTM disease.

These findings should be taken into account when designing clinical trials for novel tuberculosis vaccines in developing countries, where exposure to environmental NTM might affect both accuracy of the diagnostic endpoint and immunogenicity of the trial vaccine.

## Supporting Information

Ethics Committee Approval Document (0.09 MB PDF)
