# Supplementary material for: Isolation of Non-Tuberculous Mycobacteria in Children Investigated for Pulmonary Tuberculosis
Source: PLoS One. 2006 Dec 20;1(1):e21. doi: 10.1371/journal.pone.0000021 (PMC1762386; doi:10.1371/journal.pone.0000021)
Supplement: Ethics Committee Approval Document — (0.09 MB PDF) [file pone.0000021.s001.pdf]

501

UNIVERSITY OF CAPE TOWN

Research Ethics Committee
Faculty of Medicine
Anzio Road, Observatory, 7925
Queries : Mandisa Tani
Tel : (021) 406-6492 Fax: (021) 406-6390
E-mail : Mtani@curie.uct.ac.za

12/02/2001

REC REF: 271/2000

Prof G Hussey
Paediatrics
Red Cross

Dear Prof Hussey

A RANDOMISED, CONTROLLED TRIAL COMPARING EFFICACY OF PERCUTANEOUS AND INTRADERMAL VACCINATION WITH JAPANESE (TOKYO) 172 BCG IN THE PREVENTION OF TUBERCOLOSIS IN INFANTS VACCINATED AT BIRTH

Thank you for your application submitted to the Research Ethics Committee on the 09 November 2000.

I have pleasure in informing you that the above study (Including Protocol dated 02 November 2000, English, Afrikaans and Xhosa Patient Informed Consent Forms, English, Afrikaans and Xhosa Consent Forms for TB Case Verification including HIV Testing, English, Afrikaans and Xhosa Consent Forms for Initial Enrolment in Case Control Study, Copy of Declaration by Sponsor Form and Copy of MCC Approval) has been **formally approved** on the 09 February 2001.

Included is a list of Research Ethics Committee Members who have formally approved your protocol.

Please quote the above Reference number in all correspondence.

Yours sincerely

PROFESSOR CR SWANEPOEL
CHAIRPERSON
